# Ulnocarpal Abutment Syndrome With Triquetrum Degeneration

**DOI:** 10.7759/cureus.59585

**Published:** 2024-05-03

**Authors:** Anthony T Joseph, Ava Toluie, Peter A Hrehorovich

**Affiliations:** 1 Orthopedic Surgery, Lake Erie College of Osteopathic Medicine, Bradenton, USA; 2 Pediatrics, Lake Erie College of Osteopathic Medicine, Bradenton, USA; 3 Diagnostic Radiology, AdventHealth Sebring, Sebring, USA

**Keywords:** ulna bone, tfcc, triangular fibrocartilage complex injuries, uas, arthrogram, ulnar sided wrist pain, triquetrum degeneration, positive ulnar variance, ulnar impaction syndrome, ulnocarpal abutment syndrome

## Abstract

We present the case of a 47-year-old patient with a congenital positive ulnar variance and elucidate its effects on nearby structures in relation to ulnocarpal abutment syndrome (UAS). While magnetic resonance imaging (MRI) helped to identify soft tissue changes in the wrist, the use of an arthrogram, in this case, allowed for a more comprehensive and detailed analysis of the ligaments and soft tissues. Image findings included a complex degenerative tear of the disc of the triangular fibrocartilage (TFCC), a degenerated triquetrum, and partial tears of the scapholunate and lunotriquetral ligaments. Mild dorsal angulation of the lunate was noted, representing dorsal intercalated segmental instability (DISI), suggesting scapholunate ligament injury. Palmar classification was utilized to classify the extent of the TFCC injury as Type IIE. This case shines a light on the presentation of UAS in a patient that was not the usual demographic affected by this pathology, as well as their UAS affecting the triquetrum rather than the more commonly associated lunate.

## Introduction

The causes of ulnar-sided wrist pain are often vague and multifactorial with many etiologies, one of which is ulnocarpal abutment syndrome (UAS), also known as ulnar impaction syndrome. UAS can result from chronic degenerative changes or acute traumatic injuries. Young athletes are often the culprit for acute injury-induced UAS, as they frequently place stress on the ulnocarpal joint through axial loading and ulnar deviation [1]. Studies have also shown increasing age to be associated with a higher prevalence of UAS, representing chronic degenerative changes [2].

One of the most important predisposing factors for UAS is positive ulnar variance characterized by the distal articular surface of the ulna being greater than 2.5 mm in height relative to the radius at the distal radioulnar joint (DRUJ) in a neutral pronation-supination position [3]. The normal distribution of axial loading at the DRUJ is approximately 82% radius and 18% ulna [4]. A positive ulnar variance often results in UAS due to an alteration of the loading mechanics on the ulnocarpal joint, placing impaction stress on the surrounding structures. These structures include the lunate bone, triquetrum bone, triangular fibrocartilage complex (TFCC), lunotriquetral ligament, and scapholunate ligament [5]. The TFCC, located between the ulnar head, triquetrum, and lunate, serves as a stabilizer for the ulnar head and can be severely degenerated in positive ulnar variations, both acutely and chronically [6]. The impaction stress to the TFCC can cause central maceration and degeneration of its components as described in the Palmer classification for TFCC injury, which is broken down into traumatic (Type I) or degenerative (Type II) injuries [6]. This case report focuses on a Palmar Type IIE TFCC injury secondary to UAS.

## Case presentation

In this case, a 47-year-old patient presented to the hospital with recurrent right-sided ulnar pain for three months that was sharp in quality and worsened with axial loading during the transfer from seating to standing as well as transfers in and out of bed. The patient noted that her pain was a constant 5/10 intensity at rest, and with movement it worsened to a 7/10, referring to zero being no pain and ten being the worst pain possible. The pain was present during the day and throughout the night at baseline. The patient had tried oral ibuprofen and topical diclofenac, wrist splinting, and physical therapy, as well as a steroid injection of the right wrist with no minimal resolution of the pain. The patient had a pertinent history of seronegative rheumatoid arthritis treated with abatacept and denies any acute fall or trauma. The patient also had a past medical history of bilateral carpal tunnel syndrome, type II diabetes mellitus, hypothyroidism, and Sjogren’s disease. A physical exam revealed ulnar-sided wrist pain over the dorsum of the right hand, as well as pain with ulnar deviation and forceful pronation of the wrist. The patient had normal pinch and grip testing. The patient had a positive Tinel’s sign with paresthesias extending into the thumb and pointer finger. An initial X-ray of the wrist was unable to be transferred from the rural outpatient orthopedic office to the community hospital. The patient was being followed at an outpatient orthopedic office and was scheduled for an arthrogram of the right wrist and subsequent magnetic resonance imaging (MRI).

**Figure 1:**
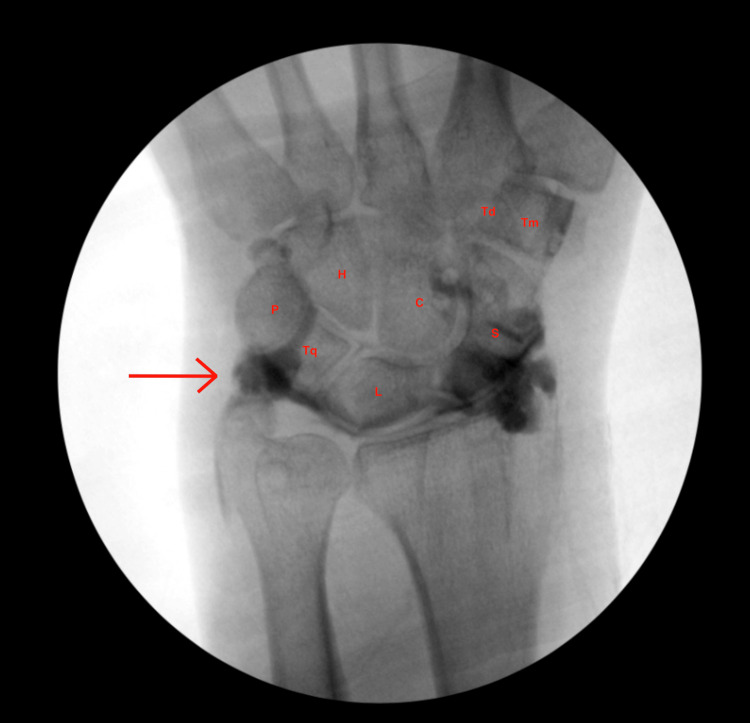
Anteroposterior view fluoroscopic wrist arthrogram of a single radiocarpal compartment intra-articular injection. A positive ulnar variance is noted. Opacification of the midcarpal joint suggests tears of the scapholunate ligament and/or lunotriquetral ligament. There is no frank extravasation of contrast into the DRUJ. The presence of contrast in the ulnocarpal joint suggests that the integrity of the TFCC and synovium of the ulnocarpal joint was compromised. TFCC = triangular fibrocartilage complex, DRUJ = distal radioulnar joint, S = Scaphoid, L = Lunate, Tq = Triquetrum, P = Pisiform, Tm = Trapezium, Td = Trapezoid, C = Capitate, H = Hamate.

A fluoroscopy-guided arthrogram of the right wrist was done utilizing a single intra-articular compartment injection of the radiocarpal joint. An anteroposterior view of the fluoroscopic wrist arthrogram demonstrated an irregular TFCC with contrast in the mid-carpal row suggestive of scapholunate and/or lunotriquetral ligament tears (Figure 1). The contrast was injected into the radiocarpal compartment, and the presence of contrast in the ulnocarpal joint space was suggestive of a tear in the TFCC and synovium surrounding the ulnocarpal joint (Figure 1).

**Figure 2:**
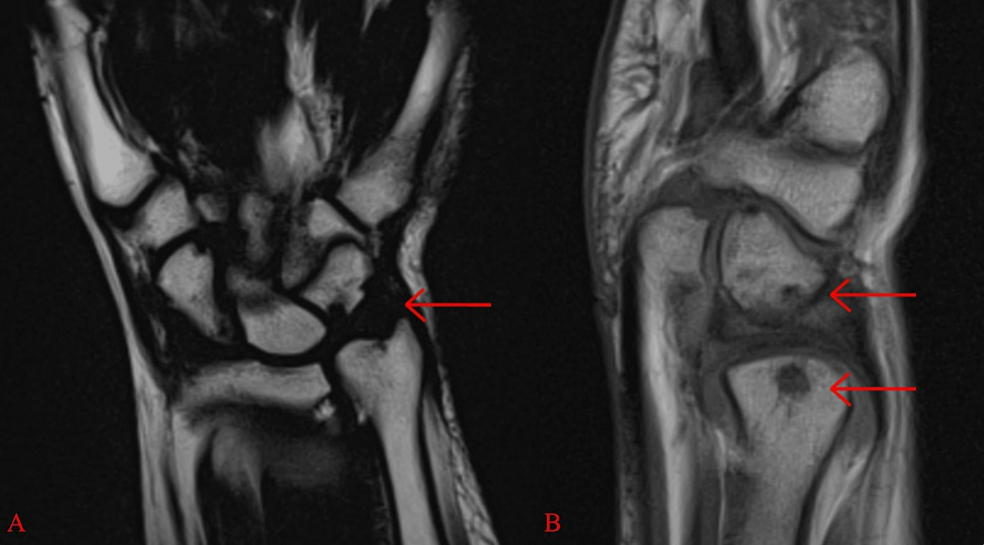
(A) T1-weighted coronal imaging reveals a positive ulnar variance of 2.9 mm and degenerative subcortical cyst formation of the proximal cortex of the triquetrum and the distal ulna. (B) T1-weighted sagittal imaging at the level of the triquetrum reveals moderately advanced degenerative signal changes of the distal ulna and the triquetrum due to chronic ulnotriquetral impaction. TFCC = triangular fibrocartilage complex

While the wrist was in a neutral pronation-supination position, T1-weighted coronal imaging revealed a 2.9 mm positive ulnar variance that induced degeneration of the triquetrum (Figure 2A). Multiple subcortical cysts were noted in the distal ulna and triquetrum bone (Figure 2A, 2B). Chronic ulnotriquetral impaction also caused moderately advanced degenerative signal changes of the triquetrum and distal ulna (Figure 2B). These subcortical cysts and advanced degenerative signal changes indicate ulnocarpal arthritis, which was used to classify the TFCC tear as Palmar Type IIE.

**Figure 3:**
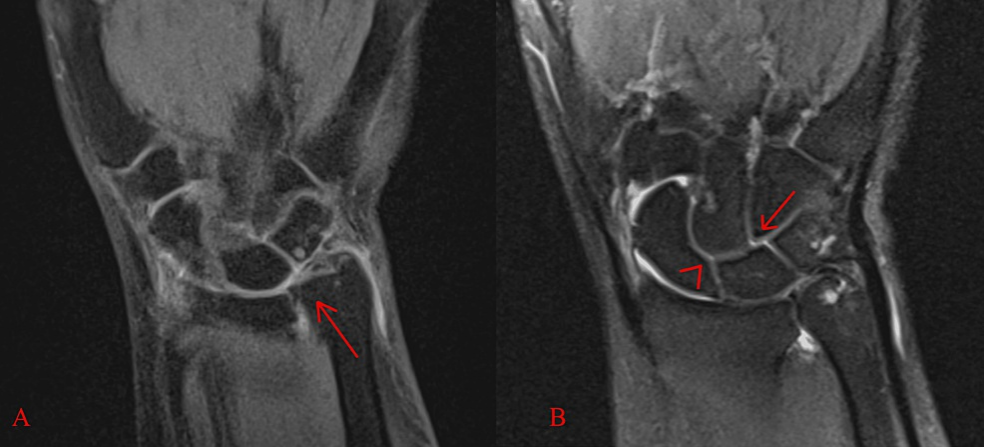
(A) Coronal fat saturation sequences reveal a complex degenerative tear of the disc of the TFCC. Additionally, high signal-intensity contrast is noted in the DRUJ, representing a tear. (B) The coronal fat saturation image reveals a small focus of intra-articular hyperintense contrast just distal to the scapholunate (arrowhead) and lunotriquetral (arrow) joint spaces, suggestive of partial tears. There is no abnormal widening of the lunotriquetral or scapholunate joint spaces. TFCC = triangular fibrocartilage complex, DRUJ = distal radioulnar joint

A coronal fat-suppressed T2 image of the wrist displayed thinning and maceration of the TFCC body and no abnormal widening of the lunotriquetral and scapholunate joint spaces (Figure 3A, 3B). Subtle partial tears of the scapholunate and lunotriquetral ligaments were appreciated due to the presence of hyperintense intra-articular contrast in the scapholunate joint space and distal to the lunotriquetral joint, respectively (Figure 3B).

**Figure 4:**
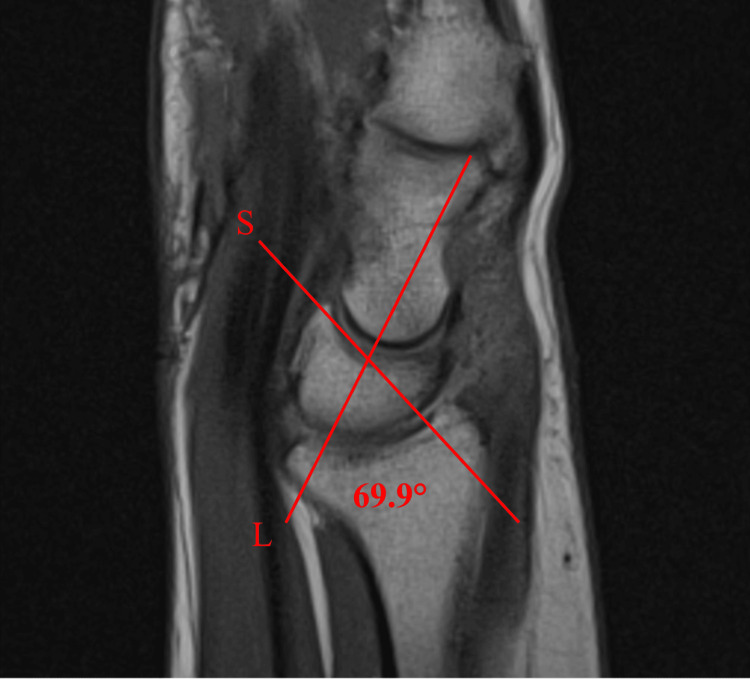
T1-weighted sagittal imaging shows a mild dorsal tilt of the lunate and mild DISI, which suggests scapholunate ligament injury/partial tear. The "S" line represents the axis of the scaphoid bone. The "L" line displays the axis of the lunate bone. Together, they create a scapholunate angle of 69.9 degrees. DISI = dorsal intercalated segment instability, S = scaphoid axis, L = lunate axis.

T1-weighted sagittal imaging of the right wrist further demonstrated a dorsally angulated lunate with a scapholunate angle of 69.9 degrees (Figure 4). This is suggestive of dorsal intercalated segmental instability (DISI) from a possible scapholunate ligament tear (Figure 4).

Due to the extent of the TFCC tear and involvement of the surrounding structures, the patient was scheduled for an arthroscopic wafer procedure, which consists of resection and debridement of the TFCC. During the patient’s right wrist arthroscopy, the scapholunate and lunotriquetral ligaments were probed, and no tears were visible. Both the volar radial and ulnar ligaments were intact. A partial synovectomy was performed, and the TFCC was observed to be severely torn and degenerated by the positive ulnar variance. The TFCC was debrided back to the stable volar and dorsal roots, and the hypertrophic capsule was debulked. The patient was scheduled for outpatient follow-up and physical therapy following the procedure.

## Discussion

UAS has traditionally been described in the presence of positive ulnar variance, but ulnar neutral and ulnar negative wrists have also been shown to contribute to this pathology [7]. The presence of a positive ulnar variance and ulnar styloid impaction primarily leads to degenerative and sclerotic changes to the lunate bone [5]. Furthermore, in a study done on UAS looking at magnetic resonance weighted imaging, abnormal signal intensity was found in the lunate of 87% of wrists, in the triquetrum of 43% of wrists, and in the ulnar head of 10% of wrists [8]. This further supports that in the majority of patients, the lunate bone is affected.

The patient's central wear of the TFCC, presence of subcortical cysts, osteoarthritis, and partially torn scapholunate and lunotriquetral ligaments help classify the TFCC injury as Palmer Type IIE pre-operatively. Palmer classification can be used to guide surgical management of the TFCC in patients with UAS. This classification is based on 1) identifying injury to the TFCC and its vascular and avascular regions and 2) identifying the presence of an intact lunotriquetral ligament [7]. Application of this classification has led to increased positive clinical outcomes, pain-free ratings, and lower scores on the disability of the arm, shoulder, and hand (DASH) questionnaire [9].

In instances like this case, chronic degenerative changes led to central maceration of the TFCC, as well as degeneration and sclerosis of the triquetrum, rather than the lunate. Additionally, preoperative imaging suggested possible scapholunate and lunotriquetral ligamentous tears; however, they were not visible during the arthroscopic procedure. It is possible that the tears were too small to be appreciated and still allowed for extravasation of contrast. Dorsal instability of the lunate was also observed, further supporting scapholunate ligament disruption, with an angle of 69.9 degrees (normal = 30-60 degrees) [10]. In this patient, the DISI may have allowed for preferential axial loading of the triquetrum rather than the lunate. The loss of anatomical alignment in the ulnar-sided carpal bones suggests that this positive ulnar variance will lead to further degeneration and decompensation of the proximal and distal carpal rows over time. Progressive worsening of the integrity of the scapholunate and lunotriquetral ligaments is also a possible outcome.

When conservative management fails, the wafer procedure has traditionally been used to treat patients with painful UAS and central perforation of the TFCC [11, 12]. The wafer procedure shows promising results in patients with UAS, particularly with significant improvement in grip strength [13]. In patients who have persistent pain after arthroscopic resection, ulnar shortening osteotomy is a successful subsequent procedure [12].

## Conclusions

Arthrogram and MRI provide substantial confirmatory and diagnostic evidence of UAS in patients presenting with ulnar-sided wrist pain, as various pathologies can lead to the presenting symptoms. The height of the distal ulna, stability of the carpal ligaments, and width of the joint spaces all contribute to normal wrist mechanics. Elucidation of this patient’s wrist pain via arthrogram and MRI identified a congenital positive ulnar variance that led to maceration of the TFCC, chronic degenerative changes to the triquetrum bone, and dorsal instability of the proximal carpal row. The effect that a positive ulnar variance can have on its surrounding anatomy allows for the implementation of the Palmar classification, which can help guide treatment measures.
